# Supplementary material for: Effectiveness of High-Intensity Laser Therapy Plus Ultrasound-Guided Peritendinous Hyaluronic Acid Compared to Therapeutic Exercise for Patients with Lateral Elbow Tendinopathy
Source: J Clin Med. 2022 Sep 20;11(19):5492. doi: 10.3390/jcm11195492 (PMC9572841; doi:10.3390/jcm11195492)

Supplementary Table S1: Linear Mixed Model, variation of hand-grip Peak strength during the follow-up according to treatment.

			Model A	Model B	Model C
Initial status	Intercept	γ_{00}	112.77±1.36 ***	93.28±1.16***	94.11±1.92***
	Treatment	γ_{01}			-2.45±2.42
Rate of change	Intercept (tempo)	γ_{10B}		Reference	Reference
		γ_{10T1}		23.28±1.02***	17.36±1.39***
		γ_{10T2}		25.53±1.28***	18.88±1.92***
		γ_{10T3}		30.26±1.63***	25.53±2.58***
		$\gamma_{11\ BHyA+HILT}$			Reference
		$\gamma_{11\ T1HyA+HILT}$			11.21±1.89***
		$\gamma_{11\ T2HyA+HILT}$			12.06±2.94***
		$\gamma_{11\ T3HyA+HILT}$			7.57±3.26*
Level 1	Within person	δ^2_e	210.81±18.49 ***	33.12±3.35***	26.94±2.73***
Level 2	In initial status	δ^2_0	68.09±20.70 ***	51.27±18.10**	61.79±18.59***
	In rate of change	δ^2_1		11.88±3.14***	11.76±2.99***
	Covariance	δ_{01}		-4.08±5.78	-6.62±5.76
		ρ	24.5	60.7	69.7
		R^2_e		84.3	87.1
		AIC	2675.7	2290.1	2181.0

*: < 0.05 – 0.01, **: 0.009 – 0.001, *** < 0.001

Supplementary Table S2: Linear Mixed Model, variation of hand-grip mean-strength during the follow-up according to treatment.

			Model A	Model B	Model C
Initial status	Intercept	γ_{00}	102.58±1.44 ***	78.37±1.16***	77.59±1.37***
	Treatment	γ_{01}			-0.09±2.16
Rate of change	Intercept [illegible]	γ_{10B}		Reference	Reference
		γ_{10T1}		40.24±1.78***	18.33±1.61***
		γ_{10T2}		31.72±1.47***	23.70±2.15***
		γ_{10T3}		30.26±1.63***	35.83±2.83***
		$\gamma_{11\ BHyA+HILT}$			Reference
		$\gamma_{11\ T1HyA+HILT}$			17.24±2.20***
		$\gamma_{11\ T2HyA+HILT}$			14.49±2.81***
		$\gamma_{11\ T3HyA+HILT}$			6.49±3.60
Level 1	Within person	δ^2_e	335.10±29.21***	54.67±5.42***	38.26±3.86***
Level 2	In initial status	δ^2_0	57.30±22.79***	19.90±16.03	31.90±15.20*
	In rate of [illegible]	δ^2_1		11.69±3.65***	13.56±3.62***
	Covariance	δ_{01}		3.42±5.72	-4.45±5.74
		ρ	14.5	27.0	45.7
		R^2_e		83.8	88.7
		AIC	2803.3	2338.5	2238.4

*: < 0.05 – 0.01, **: 0.009 – 0.001, *** < 0.001

Supplementary Table S3: Linear Mixed Model, follow-up variation of PRTEE-score according to treatments.

			Model A	Model B	Model C
Initial status	Intercept	γ_{00}	18.86±0.95***	63.55±0.66***	65.20±1.97***
	Treatment	γ_{01}			-0.98±1.31
Rate of change	Intercept [illegible]	γ_{10B}		Reference	Reference
		γ_{10T1}		-36.73±0.87***	-48.86±2.59***
		γ_{10T2}		-46.36±1.56***	-55.58±3.46***
		γ_{10T3}		-51.25±1.51***	-57.43±4.56***
		$\gamma_{11\ BHyA+HILT}$			Reference
		$\gamma_{11\ T1HyA+HILT}$			8.25±1.69***
		$\gamma_{11\ T2HyA+HILT}$			6.46±2.34**
		$\gamma_{11\ T3HyA+HILT}$			4.60±3.14
Level 1	Within person	δ^2_e	83.93±8.77***	22.75±2.30***	19.84±2.04***
Level 2	In initial status	δ^2_0	31.93±9.93***	20.59±9.35*	24.93±9.59**
	In rate of [illegible]	δ^2_1		11.27±2.77***	11.71±2.79***
	Covariance	δ_{01}		-11.65±4.50*	-13.63±4.63**
		ρ	27.6	50.0	56.8
		R^2_e		72.6	76.2
		AIC	2803.3	2070.8	2033.1

*: < 0.05 – 0.01, **: 0.009 – 0.001, *** < 0.001

Supplementary Figure S1: Ultra-sound image of the lateral elbow, longitudinal view. 1) humerus, 2) epicondyle, 3) head of radius, 4) extensor tendons, 5) calcific lesion of the tendons.

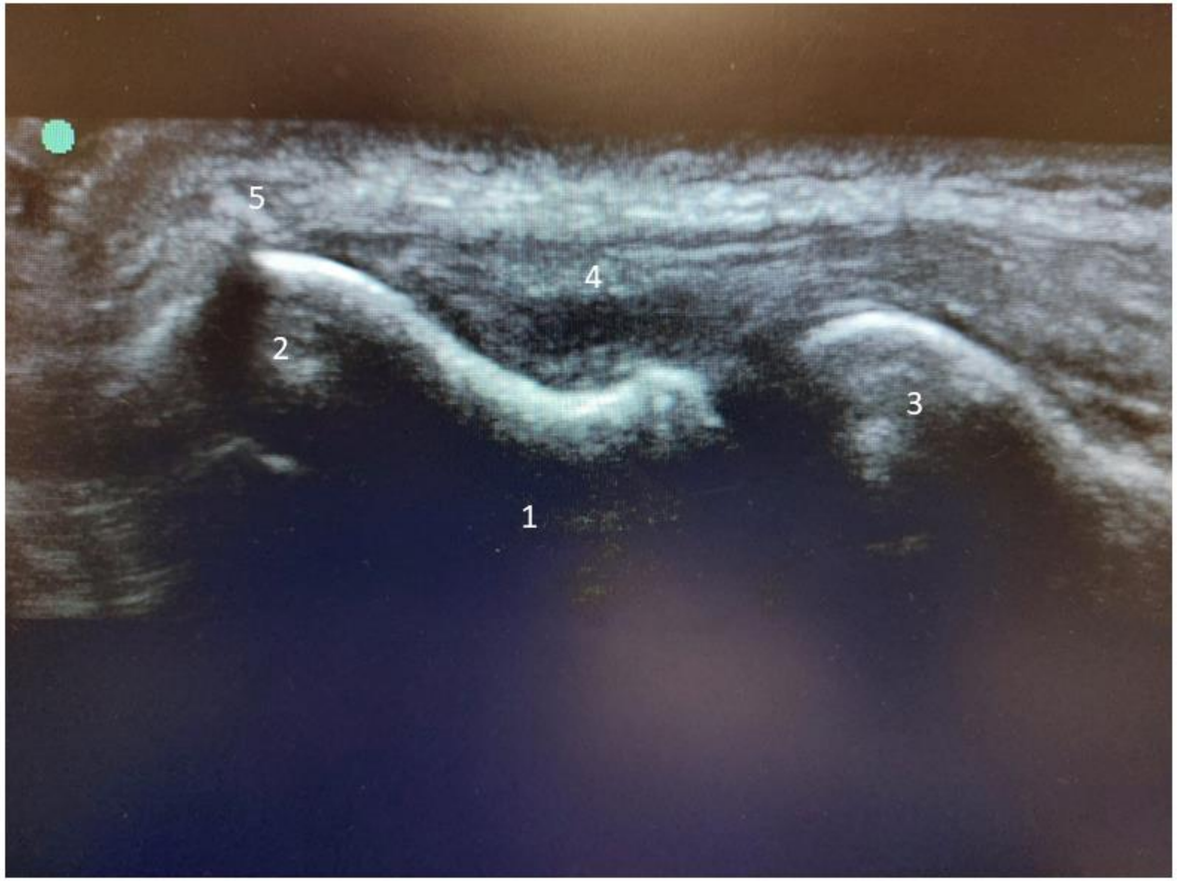

Supplementary Figure S2: Ultra-sound-guided (6-15 Mhz linear probe) Hyaluronic Acid injections in the peritendinous area of the elbow epicondyle. The injections were done using a pre-filled syringe of 20 mg in 2 ml of linear Hy-A sodium salt with a molecular weight of 500-730 kDa.

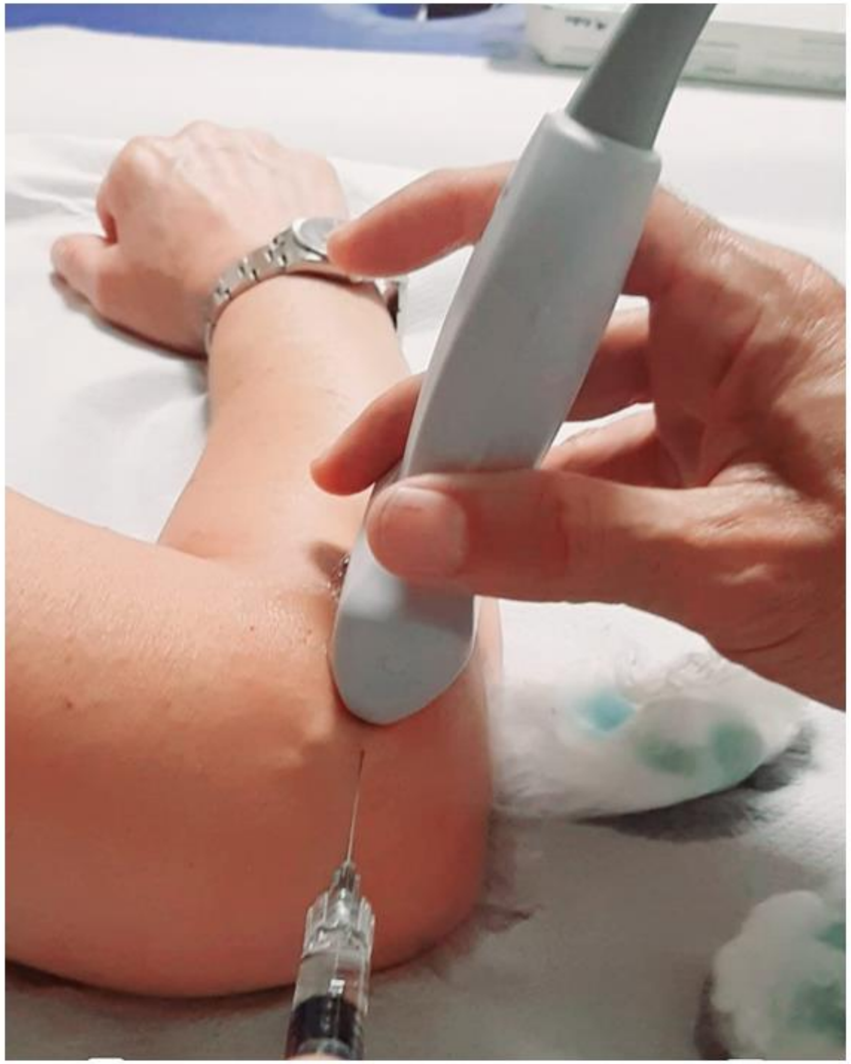

Supplement: Supplementary file 1 [file jcm-11-05492-s001.zip › jcm-1886503-supplementary.pdf]
